# Pacemaker Lead Entanglement during Interventional PFO Occlusion: Salvage Using a Sizing Balloon

**DOI:** 10.1155/2023/5586197

**Published:** 2023-12-13

**Authors:** Andreas Goldschmied, Juergen Schreieck, Michal Droppa

**Affiliations:** Department of Cardiology and Angiology, University Hospital Tübingen, Tübingen, Germany

## Abstract

We present a case of a patient with a transient ischaemic attack (TIA) likely due to paradoxical embolism through a patent foramen ovale (PFO). Her medical history included 2^nd^-degree heart block Mobitz II, which manifested with recurrent syncopes and was treated with a dual chamber pacemaker. During the interventional PFO closure procedure, we noted entrapment of the atrial pacemaker lead between the right-sided occluder disc and the interatrial septum. We were able to successfully move the lead aside using a 24 mm sizing balloon and subsequently developed the right-sided occluder disc in the correct position. In conclusion, pacemaker-lead entrapment between a PFO occluder disc and the interatrial septum can be prevented using a sizing balloon.

## 1. Introduction

In around 25% of the general population, the foramen ovale fails to close properly. This is known as the patent foramen ovale (PFO) and presents the most common congenital heart defect [1]. Although often benign, this can lead to stroke following paradoxical embolism. Interventional percutaneus PFO closure is a well-established, minimally invasive technique to close the PFO and prevent recurrent ischemia [2–4]. Devices in clinical use are deployed transvenously and consist of two metal discs which are positioned on both sides of the intraatrial septum. Although the intervention is usually safe and well established, complications can rarely occur [3]. We report a novel solution to prevent pacemaker lead entrapment by the right atrial disc of a 25 mm Gore Cardioform occluder using a 24 mm sizing balloon.

## 2. Case Report

A 65-year-old woman was admitted to our clinic due to transient paresis of her left arm. Her medical history included 2^nd^-degree heart block Mobitz II, which manifested with recurrent syncopes and was treated with a dual chamber pacemaker 12 years ago. No cardiovascular risk factors were present.

**Figure 1:**
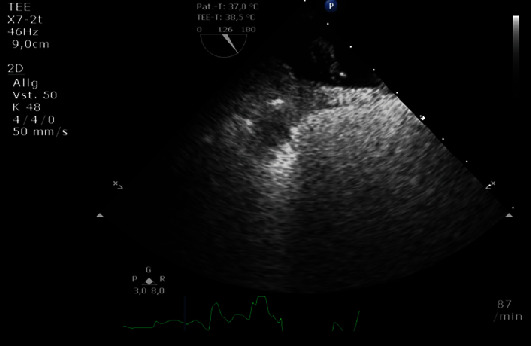
Transoesophageal echocardiography (TOE) demonstrating a large PFO with right-to-left shunt after bubble contrast injection.

A CT angiography of her head revealed no perfusion deficit or ischaemic changes. The symptoms disappeared spontaneously within a few minutes, eliminating the need for thrombolysis or mechanical thrombectomy. The diagnosis of a transient ischaemic attack was established. During the stroke workup, carotid vessel stenoses and atrial fibrillation were ruled out. Transoesophageal echocardiography (TOE) showed a large PFO with a right-to-left shunt on bubble contrast injection (Figure 1). Therefore, paradoxical embolism due to PFO was considered the cause of the ischaemic attack, and the patient was referred to our clinic for interventional PFO closure.

**Figure 2:**
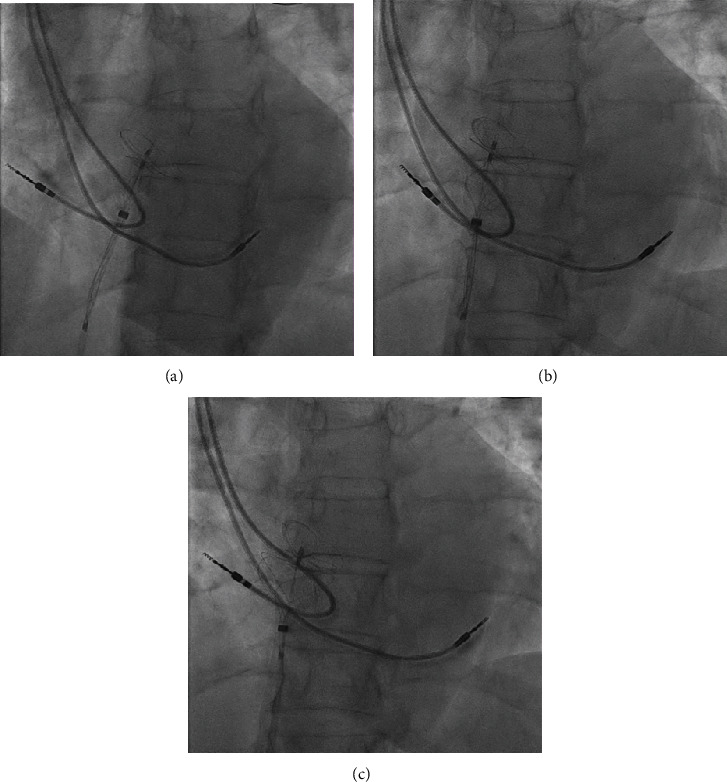
PFO occluder deployment. (a) Releasing the left-side disc of the occluder. (b) Deployment of the right-side disc with the pacemaker lead running beneath the occluder disc. (c) Entrapment of the atrial pacemaker lead between the occluder disc and intraatrial septum.

The intervention was performed under local anaesthesia and fluoroscopic guidance only. The patient received anticoagulation with 6500 I.U. of unfractionated heparin and was administered 2 g of cephazolin. The right femoral vein was selected as the access site, and a 7-F sheath was inserted. Passage to the left atrium through the PFO was achieved using a 6-F multipurpose catheter and a standard exchange wire. Subsequently, an Amplatz Super Stiff Guidewire (Meditech, Boston Scientific, Natick, MA) was inserted into the left upper pulmonary vein, and the femoral sheath was switched to an 11-F sheath for further procedural advancement. For PFO closure, a 25 mm Gore Cardioform occluder (W. L. Gore & Associates, Inc., Newark, DE) was chosen. The occluder delivery sheath was introduced into the left atrium, and the wire was removed. The left-side disc was released, and the delivery system was slightly withdrawn to press the left disc against the left side of the interatrial septum (Figure 2(a)). However, during the deployment of the right disc, we noticed that the atrial pacemaker lead became trapped between the disc and interatrial septum (Figures 2(b) and 2(c)). Unfortunately, neither wiggling nor gentle pulling or pushing manoeuvres on the delivery sheath managed to release the lead.

**Figure 3:**
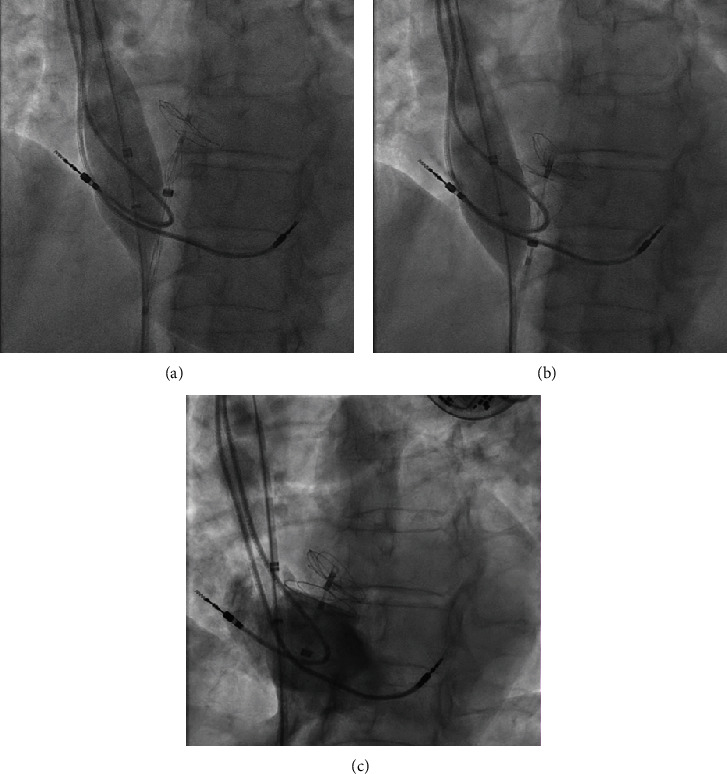
Salvage of the entrapped pacemaker lead using a sizing balloon. (a) Introduction of a 24 mm sizing balloon to the right atrium and inflation. (b) Freeing the entrapped lead by a careful pullback of the sizing balloon. (c) Confirming the correct position on fluoroscopy using a contrast agent.

To address this issue, a 7-F sheath was placed into the left femoral vein, and a standard exchange wire was advanced to the superior vena cava. A 24 mm-sizing balloon (Abbott Amplatzer) was then introduced into the right atrium and inflated in a position superior to the entrapped pacemaker lead (Figure 3(a)). With careful pullback of the sizing balloon, we managed to free the entrapped pacemaker lead, deploy the right disc, and release the occluder in the correct position. This was confirmed on fluoroscopy after the administration of a contrast agent (Figures 3(b) and 3(c)).

The day after the procedure, transthoracic echocardiography (TTE) confirmed the correct placement of the occluder, and pacemaker interrogation demonstrated good sensing and pacing thresholds, with no change in lead impedance. A follow-up TOE was scheduled for 6 months after the intervention. Additionally, prophylaxis for endocarditis was advised for a duration of 12 months. Furthermore, we recommended dual antiplatelet therapy with aspirin and clopidogrel for 6 months, followed by aspirin as a monotherapy for further 6 months. The patient was discharged on the day after the intervention.

## 3. Discussion

Although rare, percutaneous PFO closure can be complicated by pacemaker-lead entrapment. In such cases, it is essential to free the electrode in any way possible before releasing the device, as it cannot be repositioned after the occluder is released from the catheter. Otherwise, a pacemaker lead entrapped by the right disc of the PFO device poses an additional risk of lead insulation erosion and subsequent lead dysfunction. Various methods have been reported to address this issue. Quevedo et al. used a cardiac biopsy sheath to snare and release an entrapped pacemaker lead beneath the disc of an Amplatzer occluder, while Demkow et al. resolved a similar situation using a pigtail catheter with a cut-off tip [5, 6]. Meltser et al. employed cardiac biopsy forceps to release an entrapped lead beneath a CardioSEAL device [7].

In our case report, we present a novel technique to release a trapped pacemaker lead underneath a PFO closure device. The manoeuvre was relatively straightforward from a technical standpoint. During pullback, the inflated sizing balloon made contact with the proximal portion of the atrial lead and gently pushed it aside to create space for the right disc of the occluder. This worked well because the lead ran parallel to the interatrial septum. In contrast to the aforementioned approaches using a cut-off pigtail catheter and biopsy forceps, our manoeuvre did not involve direct manipulation of the pacemaker lead. This might be associated with a lower risk of iatrogenic pacemaker lead corruption and dislocation. However, it should be noted that this technique might not be suitable for all anatomical situations. Sometimes, using other catheters such as a multipurpose catheter could also be an effective option for releasing trapped pacemaker leads before advancing the methods described above.

In our case, the pacemaker electrodes were implanted 12 years ago. Regardless of the method used, in recently placed pacemaker leads, the risk of dislocation is higher. We would prefer to postpone the PFO-closure procedure for at least several weeks after the pacemaker implantation to reduce the risk of lead dislocation.

Pacemaker lead entrapment can lead to delayed endothelialisation, may prohibit complete PFO closure, and might cause electrode erosion. An infected pacemaker lead has even been reported as a source for a paradoxical septic embolus [8]. Hence, physicians should make sure to prevent this complication during interventional PFO closure. Anatomical features such as Eustachian valves or Chiari networks might also interfere with occluder devices in a similar way. However, they are not visible in fluoroscopy unless a contrast agent is used. Therefore, echocardiographists need to pay close attention to those structures while screening patients for PFO closure, particularly since Eustachian valves might even predispose to paradoxical embolism [9]. Peri-interventional TOE might be warranted in such cases.

In conclusion, the use of sizing balloons can be an effective approach to free pacemaker leads that become entrapped between the interatrial septum and the right disc of a PFO occluder during implantation. However, each case should be carefully assessed, and alternative methods may be required depending on the anatomical considerations.

## Data Availability

This case report's underlying data are available from the corresponding author upon request.
